# Denudation of the Dental Pulp

**Published:** 1857-01

**Authors:** S. Lee Rymer


### ARTICLE VIII.

*Denudation of the Dental Pulp.* By S. LEE RYMER, ESQ.

THE record of the treatment of the following case may be of use to inexperienced practitioners:

A few days ago a young man in humble life applied to me for relief from violent suffering, caused by the fracture of a tooth in an attempt at extraction. It appeared that the patient had placed himself in the hands of a druggist to have an aching tooth extracted. The operator, however, broke the tooth into fragments, and dismissed the patient, assuring him that the gum would grow over the root, and that all would be well. After experiencing the most cruel pain for three days and nights he consulted me, when, upon examination, I found that the entire pulp of the second lower molar tooth (left side) was exposed, and greatly enlarged from inflammation. In fact, the tooth which had protected it had been cracked, just as a nut is cracked to expose its kernel. I immediately coated the pulp with *nitrate of silver*, and directed the young man to call upon me the following morning; which he accordingly did, having experienced entire relief within two hours of the application of the nitrate of silver. Upon examination, I found the inflammation sensibly abated, the surface of the pulp being completely cauterized; but as *pressure* upon the part caused pain, I applied nitric acid by means of a small pellet of cotton wool grasped in a pair of lower stump forceps, which enabled me to cause the acid to act freely on the pulp, without injury to the adjoining tooth or to the surrounding parts. The young man has ever since been altogether free from inconvenience. A more speedy method of obtaining relief might, of course, have been found in extracting the fangs of the tooth or in removing the pulp; but, in this as in most similar cases, the patient would not submit to either process.—*Ib.*

CROYDON, *July*, 1856.

